# Women of reproductive age in a tertiary intensive care unit: indications, outcome and the impact of pregnancy—a retrospective cohort study

**DOI:** 10.1186/s12905-021-01396-0

**Published:** 2021-06-19

**Authors:** Karishma P. Ramlakhan, Diederik Gommers, Carmen E. R. M. Jacobs, Khaoula Makouri, Johannes J. Duvekot, Irwin K. M. Reiss, Arie Franx, Jolien W. Roos-Hesselink, Jérôme M. J. Cornette

**Affiliations:** 1 Department of Cardiology, Erasmus MC, University Medical Center Rotterdam, PO Box 2040, 3000 CA Rotterdam, The Netherlands; 2 Department of Adult Intensive Care Medicine, Erasmus MC, University Medical Center Rotterdam, Rotterdam, The Netherlands; 3 Department of Obstetrics and Gynecology, Erasmus MC - Sophia Children’s Hospital, University Medical Center Rotterdam, Rotterdam, The Netherlands; 4 Division of Neonatology, Department of Pediatrics, Erasmus MC - Sophia Children’s Hospital, University Medical Center Rotterdam, Rotterdam, The Netherlands

**Keywords:** Critical care, Pregnancy, Mortality, Women’s health

## Abstract

**Background:**

To evaluate the indications for admission and mortality rates of women of reproductive age admitted to a tertiary Intensive Care Unit (ICU) and to compare the outcomes of obstetric and non-obstetric admissions.

**Methods:**

A retrospective cohort study was performed, including all women aged 17–41 years admitted to a level 3 ICU in the Netherlands, between January 1, 2000 and January 1, 2016. Primary outcome was indication for admission and mortality. Mortality, length of stay (LOS), need for mechanical ventilation and APACHE II score were compared between obstetric and non-obstetric admissions. The obstetric group was further analyzed for maternal and perinatal outcomes.

**Results:**

3461 women (median age 32 years) were included, with an overall mortality rate of 13.3%. The obstetric group consisted of 265 women (7.7%). The non-obstetric group (n = 3196) was admitted most often for cardiovascular disease (19.6%), followed by oncologic disease (15%). Mortality was the highest in women with oncologic disease (23.9%). The obstetric group had lower mortality compared to the non-obstetric group (4.9% vs. 14%, *p* < 0.001), despite higher APACHE II score (14 vs. 11, *p* < 0.001) and a higher ventilation rate (47.9% vs. 39%, *p* = 0.004). Major surgical or endovascular interventions, besides caesarean section, were performed in 46% of the obstetric group. Perinatal death occurred in 17.2% and of the surviving infants, 63.2% were born preterm and 45.1% required Neonatal Intensive Care Unit admission.

**Conclusions:**

Cardiovascular disease is the most important indication for admission and oncologic disease is associated with highest mortality in women of reproductive age. Obstetric patients constitute a small percentage of all ICU admissions in a tertiary ICU center. They have lower mortality rates than non-obstetric young female patients, despite a more severe initial presentation. Nevertheless lasting maternal morbidity and perinatal mortality and morbidity is frequent.

## Background

The average Intensive Care Unit (ICU) patient is male, > 60 years of age and has significant comorbidity and multi-organ involvement [1, 2]. Most young women of reproductive age are considered in the prime of their life. They are far less likely to require admission in an ICU unit and are therefore less studied. Very little is known on indications for ICU admission and outcome in young women of reproductive age. Obstetric patients form a particular subgroup of this population. A recent survey in Australia and new Zealand showed that obstetric patients formed 1.3% of the total ICU population and 11% of the young women of reproductive age in the ICU [3]. Pregnancy specific problems like pre-eclampsia, postpartum hemorrhage or deterioration of pre-existent conditions can be sudden and life threatening, requiring immediate intensive management and monitoring [4, 5]. Most obstetricians are less familiar or lack facilities for this critical management, requiring ICU admission for these patients. Prompt and skilled interventions can rapidly counter most of these life-threatening complications. Two large nationwide studies showed that mortality for obstetric women admitted to ICU is low (0.7–1.7%) [3, 6]. Tertiary referral centers with high case load therefore invest in Obstetric High Care Units, permitting the management of most of these life threatening problems in an obstetric setting. ICU admission is then reserved for more complicated cases requiring specific critical care expertise.

Our aim was to analyze indications for admissions and respective mortality in young women of reproductive age in a tertiary care referral ICU. We compared outcomes between obstetric and non-obstetric admissions and described maternal and perinatal outcome in this selected population.

## Methods

We performed a retrospective cohort study in the Erasmus MC, University Medical Center Rotterdam in the Netherlands, which is the regional tertiary referral center for both obstetric and intensive care medicine. The study period ranged from January 1, 2000 until January 1, 2016. The study was approved by the Erasmus MC Institutional Review Board and the need for informed consent was waived. The handling of personal data complied with the Dutch Personal Data Protection Act; data were de-identified and handled confidentially.

In addition to the regular ward for level 1 obstetric medical care, the hospital is equipped with a level 2 Obstetric High Care Unit [7]. This dedicated unit is managed round the clock on-site by a specialized obstetrical staff, including a maternal-fetal medicine specialist, neonatologist and obstetric anesthesiologist, in close collaboration with other obstetric medicine specialists and consultants. These facilities permit continuous hemodynamic monitoring and care by trained obstetric nurses. ICU are separate wards, providing level 3 care in a closed system. Most high-risk obstetric complications like severe preeclampsia, postpartum hemorrhage (PPH) or sepsis are managed in the level 2 Obstetric High Care Unit. Admission or transfer from the level 1 or 2 wards to a level 3 ICU unit is decided for clinical reasons in communication between the obstetric caregiver and the ICU physician. Criteria for ICU admission are severe multi-organ involvement, the necessity of invasive ventilation, renal dialysis or inotropic support [7, 8].

The ICU data warehouse was screened for admissions and to collect data of young women between 17 and 41 years requiring level 3 intensive care in the aforementioned time period. The obstetric group was crosschecked with datasets from the Obstetrics department, and medical records were individually reviewed to collect data. Inclusion criteria for the obstetric group were all women requiring level 3 ICU care during pregnancy or ≤ 6 weeks postpartum.

ICD-10 codes were used to determine the indications for ICU admission, which were divided in 10 subgroups: obstetric, cardiovascular, pulmonary, gastrointestinal, neurological or neurovascular, internal medicine, surgical, oncologic, congenital (all types) or other disease. Outcomes included the APACHE II score, ICU and hospital LOS, mortality within one year and need for mechanical ventilation. The APACHE II score is a prognostic model for ICU mortality and serves as a classification for the severity of disease on admission [9].

Descriptive parameters for the obstetric admissions were parity, initial level of obstetric care, mode of delivery and moment of admission in the ICU. Indications for ICU admission were defined as direct obstetric (e.g. PPH), indirect obstetric (decompensation of underlying disease due to pregnancy) and non-obstetric pathology (unrelated to pregnancy). Data on the need for mechanical ventilation and need for intervention were collected. Interventions were defined as surgical (excluding caesarean section), endovascular or invasive medical interventions such as extracorporeal membrane oxygenation (ECMO).

Perinatal outcomes included gestational age, birthweight, 5 min Apgar score, umbilical cord pH, Neonatal Intensive Care Unit (NICU) admission, respiratory support, mortality (intrauterine, intrapartum or postpartum) and composite neonatal morbidity. Composite neonatal morbidity was defined as respiratory distress syndrome, necrotizing enterocolitis, intraventricular hemorrhage, periventricular leukomalacia and/or sepsis confirmed by blood culture in neonates born at ≥ 24 weeks of gestation, which is considered the limit of viability in the Netherlands [4].

One-year mortality rate was calculated for the entire cohort and per subgroup, after which the mortality rate of the obstetric admissions was compared to the pooled mortality of the non-obstetric admissions. LOS, APACHE II score and ventilator support rate were also compared between the obstetric and non-obstetric admissions. The detailed maternal and fetal characteristics and outcomes in the obstetric group were compared between antepartum and postpartum maternal ICU admissions. Continuous data are presented as mean with standard deviation (SD), or as median (Q1–Q3) when skewed. Differences between the groups were calculated using Student’s t-tests or Mann–Whitney tests as appropriate. Categorical data are presented as percentages and compared using χ^2^ tests. Significance was set at *p* < 0.05. Statistical analysis was carried out using IBM SPSS Statistics 21.0 (IBM New York, USA).

## Results

**Fig. 1:**
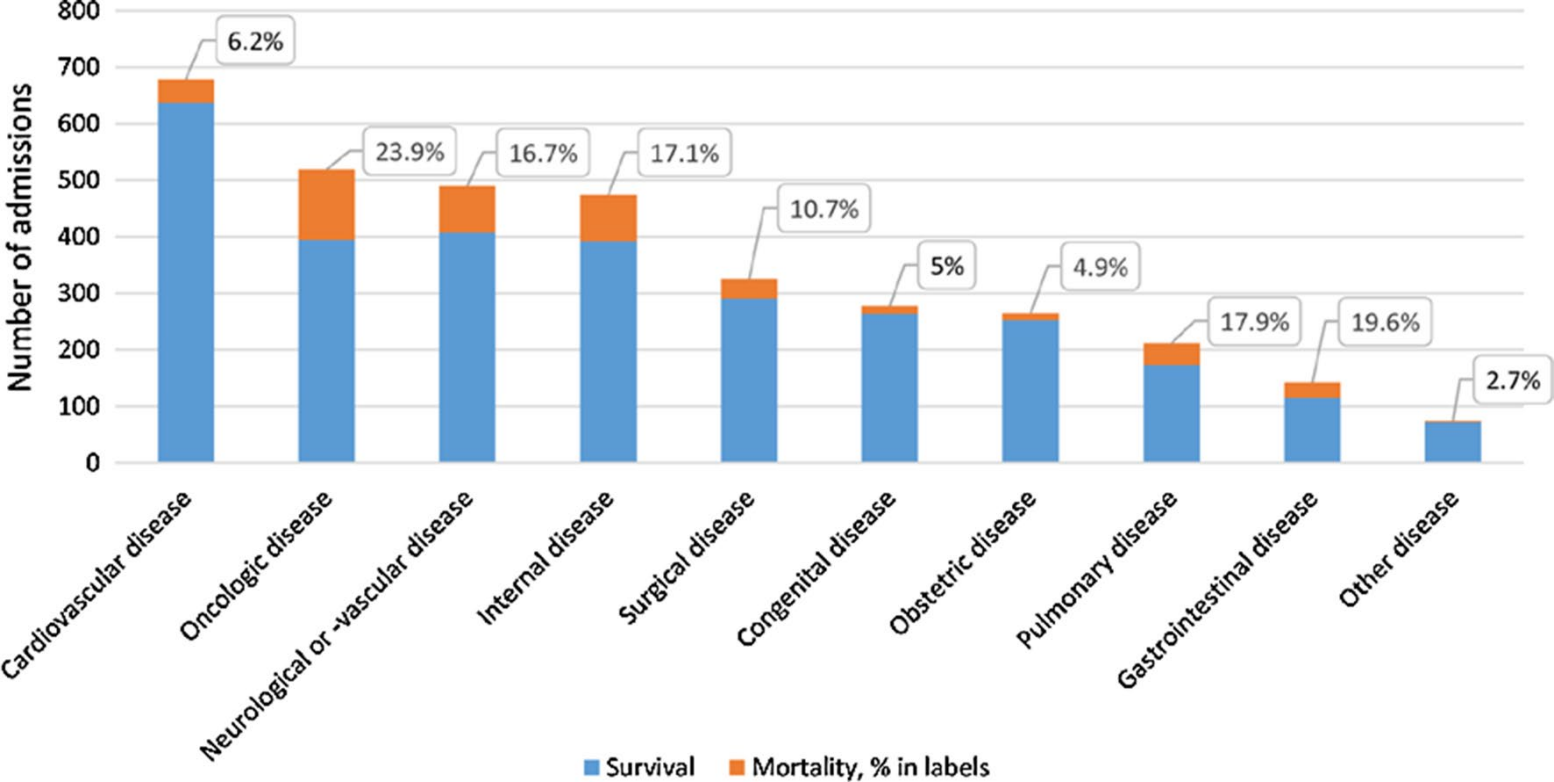
Indications for admission and associated mortality rates of 3461 young women admitted to the Intensive Care Unit from 2000 until 2015. Survival in blue, mortality in orange (% mortality within the data labels)

During the 15-year study period, 3461 female ICU admissions in 3039 women between 17 and 41 years of age were recorded. Indications for ICU admission and associated mortality rates are presented in Fig. 1. The group of admissions due to cardiovascular disease was the largest (19.6%), followed by oncologic disease (15%) and neurologic disease (14.2%). The group of obstetric admissions consisted of 265 admissions in 265 women (7.7%). The overall mortality rate of all 3461 female admissions was 13.3%. The subgroup with oncologic disease had the highest mortality rate of 23.9%, whereas the mortality rate in the obstetric group was the second lowest at 4.9%.

Table 1 presents the comparison between obstetric (n = 265) and non-obstetric admissions (n = 3196). While APACHE II score (14 vs. 11, *p* < 0.001) and use of invasive ventilation (47.9% vs. 39%, *p* = 0.004) were significantly higher, one-year mortality was lower (4.9% vs. 11%, *p* < 0.001) in the obstetric group. ICU LOS and hospital LOS were not different between obstetric and non-obstetric admissions.

**Table 1:** Outcomes of the obstetric group and the non-obstetric group

	Obstetric group n = 265	Non-obstetric group n = 3196	*p* value
Age (years)	31 (28–35)	32 (25–37)	0.195
Mortality	13 (4.9%)	446 (14.0%)	< 0.001
APACHE II score	14 (9–19)	11 (6–17)	< 0.001
Mechanical ventilation	127 (47.9%)	1247 (39%)	0.004
Hospital LOS (days)	11 (6.5–19)	10 (5–20)	0.060
ICU LOS (days)	2 (2–4)	2 (2–4)	0.343

Data in n (%) and median (Q1–Q3)

*LOS* length of stay

The obstetric baseline characteristics and admission diagnosis are further described in Table 2. Most obstetric ICU admissions (median age 31, range 17–41) took place in the immediate postpartum period (n = 198, 74.7%). Antepartum admissions (n = 67, 25.3%) occurred at a median of 28 weeks of gestation (Q1–Q3 24^1/7^–33^1/7^). Direct obstetric complications were the most common cause for ICU admission (72.5%), followed by indirect obstetric complications (21.1%), among which maternal cardiovascular disease was most frequent (9.1%). Comparing antepartum and postpartum admissions, direct obstetric causes (mainly driven by PPH) were more frequently seen in the postpartum group (58.2% vs. 77.3%, *p* = 0.003), whereas indirect obstetric causes were more frequent in the antepartum group (31.3% vs. 17.7%, *p* = 0.018). Major endovascular or surgical interventions or advanced organ support other than mechanical ventilation were performed in 46% of the obstetric group (Table 3). Uterine artery embolization was the most frequent intervention (15.8%), followed by hysterectomy (15.5%).

**Table 2:** Baseline characteristics in the obstetric group at admission to ICU

Baseline characteristics	All obstetric admissions n = 265	Antepartum admissions n = 67	Postpartum admissions n = 198	*p* value
Age, years	31 (28–35)	30 (24–33)	32 (29–35)	0.006
Nulliparity	123 (47.1%)	36 (53.7%)	87 (44.8%)	0.209
*Referred by*				
Primary care center	42 (16%)	11 (16.7%)	31 (15.8%)	0.883
Secondary care center	150 (57.3%)	40 (60.6%)	110 (56.1%)	0.554
Tertiary care center	70 (26.7%)	15 (22.7%)	55 (28.1%)	0.387
*Antepartum admissions*	67 (25.3%)	67 (100%)	–	–
Gestational age at admission, weeks	–	28 (24^1/7^–33^1/7^)	–	–
< 24 weeks	–	11 (17.5%)	–	–
24^0/7^–31^6/7^	–	35 (55.6%)	–	–
32^0/7^–36^6/7^	–	11 (17.5%)	–	–
37^0/7^–41^6/7^	–	6 (9.5%)	–	–
> 42 weeks	–	0	–	–
*Postpartum admissions*	198 (74.7%)	–	198 (100%)	–
Time between delivery and admission, days	–	–	0 (0–0)	–
*Mode of delivery*				
No delivery (< 16 weeks)^a^	18 (6.8%)	12 (17.9%)	6 (3%)	< 0.001
Vaginal delivery	87 (32.8%)	29 (43.4%)	58 (29.3%)	0.035
Caesarean section	160 (60.4%)	26 (38.8%)	134 (67.7%)	< 0.001
*Admission diagnosis*				
Direct obstetric	*192 (72.5%)*	*39 (58.2%)*	*153 (77.3%)*	*0.003*
Haemorrhage	93 (35.1%)	7 (10.4%)	86 (43.4%)	< 0.001
Hypertensive disorders	46 (17.4%)	15 (22.4%)	31 (15.7%)	0.209
Sepsis	26 (9.8%)	9 (13.4%)	17 (8.6%)	0.249
Other direct obstetric	27 (10.2%)	8 (11.9%)	19 (9.6%)	0.583
Indirect obstetric	*56 (21.1%)*	*21 (31.3%)*	*35 (17.7%)*	*0.018*
Cardiovascular	24 (9.1%)	7 (10.4%)	17 (8.6%)	0.646
Respiratory	11 (4.2%)	6 (9%)	5 (2.5%)	0.023
Cerebrovascular	5 (1.9%)	2 (3%)	3 (1.5%)	0.445
Metabolic	5 (1.9%)	2 (3%)	3 (1.5%)	0.445
Gastrointestinal	5 (1.9%)	2 (3%)	3 (1.5%)	0.445
Auto-immune	2 (0.8%)	0 (0%)	2 (1.0%)	0.409
Other indirect obstetric	4 (1.5%)	2 (3%)	2 (1%)	0.252
Non-obstetric	*17 (6.4%)*	*7 (10.4%)*	*10 (5.1%)*	*0.119*
Malignancy	4 (1.5%)	1 (1.5%)	3 (1.5%)	0.990
Trauma	2 (0.8%)	2 (3%)	0 (0%)	0.015
Other non-obstetric	11 (4.1%)	4 (6%)	7 (3.5%)	0.388

Data in n (%) and median (Q1–Q3). *p *values were calculated between antepartum admissions versus postpartum admissions

^a^No delivery was defined as miscarriage, elective or medically indicated termination of pregnancy or ectopic pregnancy

**Table 3:** Maternal morbidity

Interventions	Obstetric ICU admissions n = 265
Medical intervention only^a^	143 (54%)
Uterine artery embolization (UAE)	48 (15.8%)
Hysterectomy	41 (15.5%)
Laparotomy	27 (10.2%)
Neurosurgery (including endovascular coiling)	9 (3.4%)
Gastrointestinal surgery (e.g. colostomy creation)	9 (3.4%)
Cardiothoracic surgery	5 (1.9%)
Urological surgery	2 (0.8%)
Limb amputation	2 (0.8%)
Liver transplant	2 (0.8%)
ECMO	5 (1.9%)
Haemodialysis	14 (5.3%)
Use of vasopressors or inotropes	46 (17.1%)
Mechanical ventilation	127 (47.9%)

Data in n (%)

*ECMO* extracorporeal membrane oxygenation

^a^Medical intervention only was defined as no surgical or endovascular intervention or advanced organ support, excluding mechanical ventilation

Perinatal data was unavailable for 26 pregnancies, leading to exclusion in the analysis. There were 18 twin pregnancies in remaining 239 pregnancies, and therefore outcome is described for 257 infants in Table 4. Before the limit of viability (24 weeks of gestation), there were 6 (2.3%) spontaneous intrauterine deaths and 4 (1.5%) terminations of pregnancy for severity of maternal disease. After the limit of viability, there were 26 (9.9%) spontaneous intrauterine deaths and one (0.4%) termination of pregnancy. There were 8 (3.1%) neonatal deaths during or after delivery, making the total mortality rate 17.2%. Median gestational age at delivery and birth weight were 35 weeks and 2215 g. Preterm delivery occurred in 157 (63.2%) pregnancies, 112 (40.7%) of which were medically indicated preterm deliveries (iatrogenic). NICU admission was required in 45.1% of all surviving neonates, for a median duration of 8 days (range 0–177). Mechanical ventilation was needed in 34.1%, cardiopulmonary resuscitation was performed in 5.1% and composite neonatal morbidity was reported in 29.1% of all neonates. Mortality was higher and gestational age at birth and birth weight were lower among the infants of women admitted during pregnancy, compared to women admitted postpartum (43.1% vs. 18%, *p* < 0.001).

**Table 4:** Neonatal outcome

	All admissions n = 257	Antepartum admissions n = 59	Postpartum admissions n = 198	*p* value
Male	48.2%	52.8%	47.9%	0.524
Female	51.8%	47.2%	52.1%	
Gestational age at birth, weeks	35 (30^2/7^–38^1/7^)	31^4/7^ (27^4/7^−36^6/7^)	36 (31^4/7^−38^4/7^)	< 0.001
< 24 weeks	5.6%	12.5%	2.8%	
24^0/7^–31^6/7^	29.8%	41.1%	25%	
32^0/7^–36^6/7^	27.8%	23.2%	28.9%	
37^0/7^–41^6/7^	35.5%	23.2%	41.7%	
> 42 weeks	1.2%	0%	1.7%	
Spontaneous preterm birth	10.9%	16.9%	10.1%	0.150
Iatrogenic preterm birth	40.7%	54.2%	40.4%	0.060
Birth weight, grams	2215 (1377–3128)	1770 (1045–2780)	2510 (1510–3230)	< 0.001
Apgar at 5 min	9 (7–10)	8 (6–10)	9 (7–10)	0.234
Umbilical pH	7.26 (7.20–7.31)	7.26 (7.21–7.31)	7.26 (7.19–7.31)	0.729
Mortality	17.2%	43.1%	9.7%	< 0.001
Resuscitation	5.1%	11.1%	3.4%	0.057
Mechanical ventilation	34.1%	50%	31.2%	0.039
NICU admission	45.1%	50%	45%	0.600
Composite neonatal morbidity^a^	29.1%	41.2%	26.8%	0.100

Data in percentages and medians (Q1–Q3). *p *values were calculated between antepartum and postpartum maternal ICU admission

*NICU* neonatal intensive care unit

^a^Composite neonatal morbidity was defined as the occurrence of respiratory distress syndrome, necrotizing enterocolitis, intraventricular hemorrhage, periventricular leukomalacia and/or sepsis

## Discussion

The mortality rate of young women after ICU admission in a tertiary care center varies strongly between different indications for admissions, of which cardiovascular disease is the most frequent. Women admitted during pregnancy or postpartum have a lower mortality rate as compared to non-obstetric patients in a similar age group (4.9% vs. 14%, *p* < 0.001), despite higher APACHE II scores and a higher rate of mechanical ventilation.

The average Dutch ICU 1-year mortality rate is 22.6%, but the average ICU population is predominantly male, > 60 years old and has a median APACHE III score of 51 [10]. In our study young women have a better prognosis (mortality rate 13.3%), although this seems to depend on admission diagnosis. Cardiovascular disease was the most prevalent diagnosis and was associated with a relatively low mortality of 6.2%. The mortality rate in oncological patients was higher (23.9%) as may be expected, but other subgroups also had markedly high case fatality rates (such as 19.6% for pulmonary disease). A possible explanation for the low mortality in cardiovascular disease might be the low-threshold use of intensive care facilities for hemodynamic monitoring of cardiac patients.

Women admitted to an ICU during pregnancy or postpartum have a lower mortality rate as compared to non-obstetric patients in a similar age group (4.9% vs. 14%, *p* < 0.001), despite higher APACHE II scores and a higher rate of mechanical ventilation. Relatively favorable outcome for obstetric admissions is in line with a single previous study on the subject (3.1% vs. 19.6%) [11]. The favorable outcome for the obstetric group may be partly explained by the direct obstetric causes and many pregnant women being previously physically healthy. Moreover, the physiological adaptations during pregnancy are evolutionary designed to limit blood loss and create additional reserves [12]. Instead of being a risk factor for adverse outcome, pregnancy might actually offer some physiological protection to the mother in critical illness. Additionally, there may be a high index of suspicion and carefulness in treating pregnant patients, which may create a low threshold for ICU admissions for less complex indications than in the non-obstetric group [7].

However, this is not evident from the APACHE II score. In contrast to mortality, the obstetric group has a higher APACHE II score than the non-obstetric group. The APACHE II score has previously been observed to overestimate mortality in obstetric populations, often explained by the altered physiological ranges in pregnancy [11, 13]. However, the APACHE II normal ranges are often broad enough (heart rate 70–109; mean arterial pressure 70–109; respiratory rate 12–24) that even the physiologically altered values during pregnancy do not easily fall into the abnormal range [9, 14–16]. Other APACHE II parameters, such as the Glasgow Coma Score, are even more unlikely to be significantly influenced by pregnancy-induced changes. Recently, a pregnancy-specific risk model has been developed but this is not yet implemented in clinical care and cannot be used to compare obstetric and non-obstetric ICU admissions [6].

We found higher mechanical ventilation rates in the obstetric group as compared to the non-obstetric group. Particular care for respiratory complications should be taken in obstetric ICU care. The gravid uterus elevates the diaphragm, which combined with increased oxygen consumption and carbon dioxide production results in a decreased functional residual capacity [14].

The obstetric mortality rate (4.9%) in this study is higher than reported by the two largest studies on obstetric ICU admissions (0.7–1.7%), based on nationwide databases [3, 6]. The difference in outcomes illustrates the higher complexity of the patient population in a tertiary referral center, as compared to a nationwide sample. Additionally, there was a high threshold for ICU admission in obstetric patients due to the presence of a dedicated level 2 Obstetric High Care unit. The latter accounted for an additional 2006 admissions during the study period, which suggests that dedicated obstetrical critical care units can substantially reduce ICU admissions, as part of these severely ill women might in another hospital have been admitted to the ICU [17].

The largest studies of obstetric ICU admissions do not describe maternal morbidity beyond ICU resource utilization [3, 6]. We demonstrated that half of pregnant and postpartum patients required surgical or endovascular intervention, in addition to Caesarean section in 60.4%. The most frequently performed interventions were uterine artery embolization and hysterectomy, which are commonly performed because of peripartum hemorrhage. Many of the procedures that were performed in this cohort (e.g. hysterectomy, colostomy, limb amputation) could be speculated to have significant impact on a woman’s quality of life (QoL) [18]. In one study of QoL after obstetric ICU admission, 19% of participants reported decreased QoL even after a median ICU LOS of 22 h, which is considerably shorter than in our population [18]. The impact on QoL might be greater in a more severely ill population and warrants further research, which should include predictors of decreased QoL.

Perinatal death (17.2%) is frequent, which is in line with earlier reports describing 13.6–34% perinatal mortality in critically ill mothers [4, 19, 20]. Perinatal mortality among women admitted antepartum was higher than in those admitted postpartum, which may be associated with the (often iatrogenic) higher rate of premature births [21]. Additionally, the direct obstetric complications (such as PPH) seen predominantly in the postpartum admissions do not always affect the child. For antepartum admissions, extending the pregnancy duration may improve fetal outcome, but may also be detrimental to the mother and exposes the fetus to a potentially unfavorable environment created by maternal illness, such as acidosis or placental insufficiency [22]. The surviving infants had composite neonatal morbidity in 29.1% and NICU admission in 45.1%, which exceeds the earlier described NICU admission rate of 11–36.7% among children born from critically ill mothers in smaller cohorts [4, 19].

Limitations of this study are its retrospective nature and possible heterogeneity because of advances in treatment during the 15-year timeframe. Our tertiary referral center is not representative for all ICU’s, as we describe a high-risk population. However, this study has several strengths. We provide new insights into the causes of ICU admissions in young women, which are an often overlooked group. For obstetric admissions, we report relatively favorable mortality rates but high rates of interventions that may have lasting impact on quality of life, requiring further research. Our perinatal data may contribute to counseling on the expected fetal and neonatal complications after maternal ICU admission.

## Conclusions

In this retrospective cohort study in a tertiary center, young women are admitted to the ICU most often for cardiovascular disease and their prognosis strongly depends on the primary indication for admission. Obstetric patients constitute a minor part of ICU admissions, and their mortality rate is lower compared to other young female ICU patients. This is despite higher need for invasive ventilation and higher APACHE II score.

## Data Availability

The raw data are available from the corresponding author KPR upon reasonable request. They are not publicly available due to them containing information that could compromise research participant privacy due to the nature of the study (rare and critical events in a single center during a defined time period). Individual consent to share individual data was not obtained according to national ethics guidelines for observational retrospective studies.

## Abbreviations

ICU: Intensive Care Unit
PPH: Postpartum hemorrhage
LOS: Length of stay
ECMO: Extracorporeal membrane oxygenation
NICU: Neonatal Intensive Care Unit
SD: Standard deviation
